# Comprehensive Assessment of Pollution Sources and Health Impacts in Suburban Area of Shanghai

**DOI:** 10.3390/toxics11070552

**Published:** 2023-06-23

**Authors:** Wan Wei, Meng Wang, Qi Yuan, Zhuozhi Zhang, Xinwei Li, Shuwen Han, Yusen Duan, Qingyan Fu, Shun-Cheng Lee

**Affiliations:** 1 Department of Civil and Environmental Engineering, The Hong Kong Polytechnic University, Kowloon, Hong Kong SAR, China; wanwei@polyu.edu.hk (W.W.); yq-qi.yuan@connect.polyu.hk (Q.Y.); zhuo-zhi.zhang@connect.polyu.hk (Z.Z.); xin-wei.li@connect.polyu.hk (X.L.); shuwen.han@connect.polyu.hk (S.H.); 2 Shanghai Environmental Monitoring Center, Shanghai 200030, China; duanys@sheemc.cn (Y.D.); qingyanf@sheemc.cn (Q.F.)

**Keywords:** source apportionment, health-risk assessment, heavy metals, Shanghai, suburban area

## Abstract

Shanghai, one of China’s largest metropolises, faces significant environmental pollution challenges due to rapid economic development. Suburban areas of Shanghai are affected by both long-distance transport and local sources of pollutants. This study conducted an integrated analysis that links health-risk assessment of heavy metals and source apportionment of atmospheric constituents to distinguish the contributions of emission sources and the major sources of health risks. Source-apportionment analysis revealed that secondary sources had the greatest contribution to the local pollutants, indicating the significant influence of peripheral and long-distance transport. Health-risk assessment of Cr, Ni, As, and Cd revealed that local residents were exposed to respiratory health risks, in which Cr is the major contributor. This health risk was primarily associated with emissions from nearby industry-related sources. Our study highlights the significant effects of both long-distance transport and local source emissions on atmospheric composition and human health in large urban agglomerations. The findings can inform future efforts to develop more precise emission-reduction strategies and policy improvements to mitigate environmental pollution and protect public health.

## 1. Introduction

Air pollution is typically considered as substances excessively emitted from both anthropogenic and natural sources, and which will pose a threat to atmospheric environment, regional climate, and human health when they accumulate in sufficient concentrations [1,2,3]. Due to rapid urbanization and industrialization, many developing countries, especially China, with its large population, are experiencing air-pollution incidents similar to those previously encountered in developed countries [4,5,6]. As a representative of China’s metropolises, the cluster of cities along the route from Beijing to Shanghai is one of the most polluted areas in China, contributing 34% of the country’s PM_2.5_ emissions with only 10% of its land area [7]. Shanghai, due to its geographical location in the Yangtze River Delta (YRD), is, inevitably, subject to regional pollution caused by the intensive industrialization [8,9,10].

A series of measures have been conducted by the government to improve the regional air quality in Shanghai, including the introduction of a series of laws and regulations, strict emission standards, and the adjustment of production structure, which have significantly improved the air quality in this region. However, regionalism and its complexity are still among the most pressing problems facing Shanghai and its surrounding areas [11,12,13]. Therefore, accurately identifying and quantifying the sources of ambient PM_2.5_ is crucial for implementing effective mitigation strategies, as well as reducing the impact on public health and the environment. The positive matrix factorization (PMF) receptor model has been widely used in resolving different air-pollutant sources in the past decades [3,14,15,16,17]. However, PMF might introduce a significant amount of rotational ambiguity unique to the chosen factor resolutions [18,19]. In contrast, the multi-linear engine (ME-2) algorithm implemented within the Igor Pro software package is believed to be able to lead to a reduction in the rotational ambiguity and provide a rather unique solution [20,21]. It is also a powerful tool extensively employed for source apportionment [22,23,24].

Heavy metals, a group of elements commonly found in air pollutants, can be released into the atmosphere by various sources [25,26]. Although they might only comprise a small mass fraction of PM_2.5_, they are considered to be one of the most important components causing aerosol toxicity [27]. Exposed to certain levels of heavy metals might result in a range of health problems, including respiratory diseases, internal organ damage, and it may affect the endocrine, immune, and nervous systems [26,28]. However, previous researches on health impacts of heavy metals mainly focused on individual cities, especially large cities. Suburban areas are often overlooked as they are considered to have relatively clean air. In actuality, suburban areas are often located downwind from urban areas and are vulnerable to the long-distance transport of pollutants from surrounding cities, especially in large city agglomerations such as the YRD region.

In this study, three winter months’ worth of field observation and sampling was conducted at a suburban site of Shanghai from 2018 to 2019. Hourly time-resolved organic carbon (OC) and elemental carbon (EC) in PM_2.5_, as well as 13 elements and 3 ions, were measured. The temporal variations and the source apportionment of these pollutions are discussed. Meanwhile, the health impacts of four main heavy metals (Cr, Ni, As, and Cd) were calculated and their possible sources were estimated. Our study provides valuable insights into the characteristics and trends of air pollution in suburban areas heavily affected by remote transmission from surrounding megacities or city agglomeration. These findings can inform the development of effective policies and strategies to reduce regional air pollution and protect human health.

## 2. Materials and Methods

### 2.1. Sampling Site

**Figure 1:**
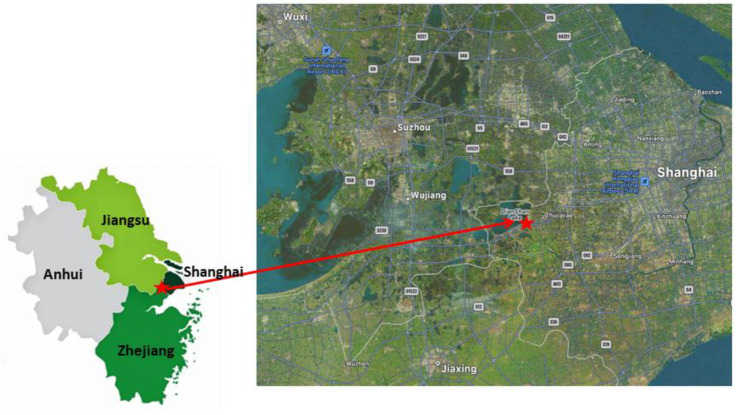
Location of sampling site (source: ^©^Apple Maps, https://developer.apple.com/maps/web/ (accessed on 15 May 2023)).

Field observation and sampling were conducted at Dianshan Lake (DSL) site, which is situated in the Qingpu District of western Shanghai and operated by the Shanghai Environmental Monitoring Center (Figure 1). This site is an ideal location for investigating the accumulation of local pollutants and long-distance transported pollution due to its geographical location, which intersects with Shanghai, Jiangsu, and Zhejiang provinces. Though it is near DSL and represents the environment of suburban Shanghai, it is considered to be affected by the industrial activities from surrounding areas, as well as the two highways beside it. Therefore, it is an ideal site to investigate the local pollutants accumulation and long-distance transported pollution [10].

### 2.2. Instruments and Measurements

In this study, ambient PM_2.5_ was detected over three winter months from November 2018 to February 2019. The mass concentrations of the samples were determined using a tapered-element oscillating microbalance monitor (TEOM, Thermo FH62C-14, Waltham, MA, USA) with a sampling flow rate of 16.7 L/min. The hourly measurement carried an uncertainty of ±1.50 mg/m^3^, and the detection limit was 0.1 μg/m^3^. Consistent with a previous study [29], the concentration of PM_2.5_ was converted to hourly means. Water-soluble inorganic ions, such as sulfate and nitrate in PM_2.5_, were measured by an online monitor for aerosols and gases (MARGA, model ADI2080, Metrohm Applikon B.V., Schiedam, The Netherlands).

Online measurements of OC and EC were conducted using a Sunset Semi-Continuous Carbon Analyzer (Sunset Laboratory, Forest Grove, OR, USA), which employs the thermal-optical transmittance method at a flow rate of 8 L/min. This method involves heating the sample to different temperatures in a controlled environment, and measuring the amount of carbon released at each temperature. By comparing the amount of carbon released at different temperatures, it is possible to differentiate between organic and elemental carbon. Hourly time-resolved analyses of OC and EC were obtained using this instrument, with detection limits of 0.2 and 0.04 μg/m^3^, respectively.

### 2.3. Assessment of Health Risks of Heavy Metals

Health risk caused by heavy metals in PM is mainly carried in three ways: ingestion, inhalation, and dermal contact [26,27,30], of which inhalation is an important route of exposure, as the pulmonary bronchus is particularly vulnerable to exposure to PM_2.5_ [31], and the heavy metals adsorbed therein are considered as a significant risk factor for lung cancer [32]. In this study, we used health-risk assessment models proposed by the United States Environmental Protection Agency (USEPA) to evaluate the health risk of four major toxic heavy-metal elements (Cr, Ni, As, and Cd) in PM_2.5_ collected from the site. The average exposure amount via the inhalation pathway of each individual in a given time span can be calculated by Equation (1):

(1) $$D_{inh}=\left(C\;\times \;\mathrm{IR}\;\times \;\mathrm{EF}\;\times \;\mathrm{DE}\right)\;/\;\left(\mathrm{BW}\;\times \;\mathrm{AT}\right)\;\;$$

in which $D_{inh}$ represents average daily dose for non-cancer risk or lifetime average daily dose for cancer risk (mg/(kg·d)); *C* represents heavy-metal element concentration in PM_2.5_ (mg/m^3^); *IR* represents respiration rate (m^3^/d), take 17.6 m^3^/day as the average for the surveyed population across different age groups according to a previous study [30], and the subsequent parameters are also taken from this study; *EF* represents exposure frequency (d/year), take the average of 255 for different age groups; *ED* represents exposure duration (d), take the average of 70 for different age groups; *BW* represents body weight (kg), take 60 kg as the average value; and *AT* represents the averaging exposure time (d), 70 × 365 d was used for both carcinogens and non-carcinogens.

The hazard quotient (HQ) for non-carcinogenic risk and incremental lifetime cancer risk (ILCR) of inhalation exposure can be calculated, respectively [33]. The equations are:

(2) $$\mathrm{HQ}=D_{inh}\;/\;RfD$$

(3) $$\mathrm{ILCR}=D_{inh}\times SF$$

in which *RfD* is the reference dose which represents an estimate of daily exposure to the human population (mg/(kg·day)), 2.86 × 10^−5^, 2.06 × 10^−2^, 3.01 × 10^−4^ and 1.00 × 10^−3^ for Cr, Ni, As, and Cd, respectively [34]; *SF* is the cancer slope factor (mg/(kg·d)), take 42, 0.84, 15.1, and 6.3 for Cr, Ni, As, and Cd, respectively [34].

### 2.4. Source Apportionment Using the Tracer-Based Approach

The ME-2 algorithm was utilized to conduct the source apportionment in this study. The receptor model was performed using the source finder tool (SoFi v6.7) [21] within the Igor Pro software package (Wavemetrics Inc., Lake Oswego, OR, USA). Species input includes 2 carbonaceous materials, 3 water-soluble inorganic ions, 13 elements, and 8 health risk indices. In order to incorporate the health-risk factors (ILCR and HQ) into the source analysis and apportionment using ME-2, equivalent conversions of the original factors were performed based on the following equations:

(4) $${\mathrm{ILCR}}_{\mathrm{conver}}=10\;/-\log\left(ILCR_{ori}\right)$$

(5) $${\mathrm{HQ}}_{\mathrm{conver}}=10\;/-\log\left(HQ_{ori}\right)$$

After the equivalent conversions, the health-risk indices of the heavy metals input in the ME-2 algorithm are at the similar magnitude as the element concentrations (ng/m^3^), avoiding the possible impact which may cause to the elemental analysis due to the direct addition of ILCR and HQ. Finally, the input matrix has a dimension of 2622 × 26. The statistical data for the input species are summarized in Table S1. The elements of the measurement uncertainties (Unc) matrix were calculated by the following equation [35]:

(6) $$Unc_{ij}=\sqrt{{\left(DL_{j}\right)}^{2}+{\left(CV_{j}x_{ij}\right)}^{2}}$$

where $DL_{j}$ is the detection limit for compound *j* ($DL_{j}$ was calculated as twice the standard deviation of the field blanks [36]); and $CV_{j}$ is the coefficient of the variation for compound *j*.

In order to identify the ideal number of source factors, a series of rigorous tests were conducted, ranging from 1 to 10 factors. For each factor, 10 different random starting points were initialized to ensure the accuracy of the results. The Q/Q_exp_ ratio, usually used to measure the explanatory power of adding factors on the variation of the input dataset, decreased continuously from 12.9 to 1.91 when the number of factors increased from 1 to 10. The final number of factors was determined by the variation of Q/Q_exp_ ratio with the number of factors. As shown in Figure S1, the Q/Q_exp_ ratio displayed a smaller decrease when it is moving from 7 to 8 factors, suggesting 7 factors are sufficient to account for the variability present in the data [37,38]. Details of the evaluation can be found in our previous study [24]. The model can well match the variation of input species, as evidenced by high correlation values between modeled output and the input values. Specifically, the correlation analysis revealed a slope of 0.91 and R^2^ of 0.96, which are presented in Figure S2.

## 3. Results and Discussion

### 3.1. Characteristics of Pollutants during the Sampling Period

**Figure 2:**
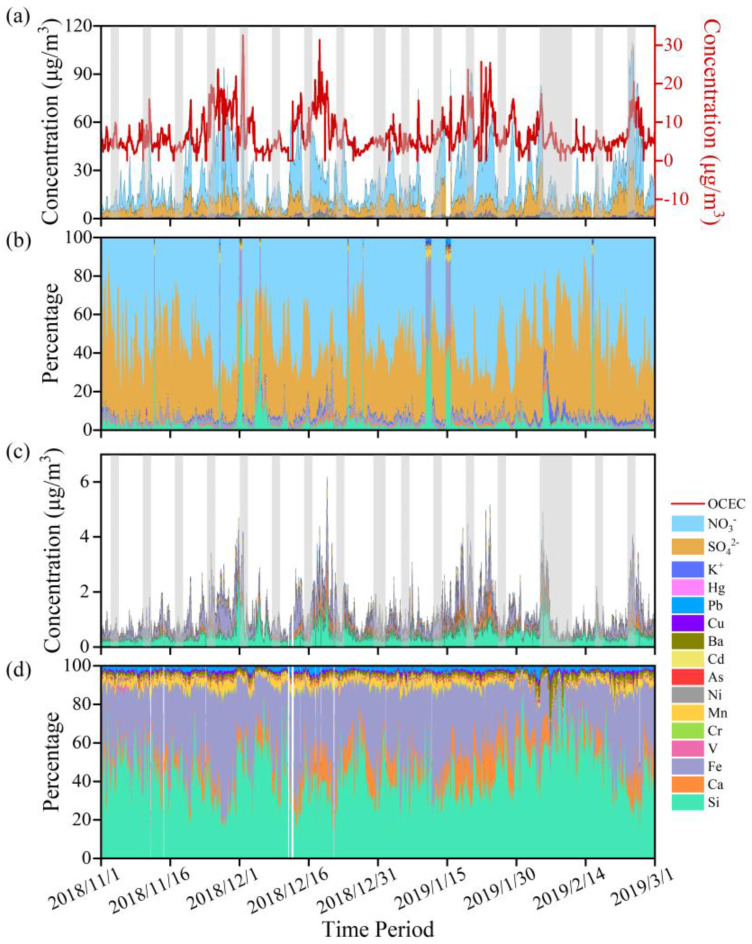
Temporal variations and distribution in all the detected pollutants (**a**,**b**), and the elements (**c**,**d**). The shaded areas represent the non-working days (weekends and legal holidays).

As shown in Figure 2, the levels of pollutants fluctuated over the period, with clear peaks seen from 25 November–1 December in 2018, 14 January–4 February in 2019, and 23–26 February in 2019. Further investigation of the surrounding events and news revealed that these peaks corresponded to the middle and late periods of the China International Automobile Expo, the China Industry Expo and other large trade fairs, the traditional Chinese Spring Festival holiday and the late period of the Lantern Festival, respectively. Meanwhile, a serious haze occurred in Shanghai from 20 to 25 November in 2018. Such observation results indicate that the DSL area, as a junction of several large cities, is affected by the transport of surrounding pollutants while being affected by local emissions. The shaded areas in Figure 2 represent the weekends and legal holidays. It can be seen that during these non-working days high concentrations of pollutants accumulated at times, revealing that population flow and travel during non-working days do have an impact on suburban air quality, but other contributing factors are also involved.

Temporal variations of all the pollutants observed are shown in Figure 2a. The total concentration of detected pollutants ranged from 11.62 to 208.42 μg/m^3^, with an average concentration of 49.55 ± 36.60 μg/m^3^. However, this average concentration far exceeded the World Health Organization latest recommended air-quality guidelines (5 μg/m^3^) [39], indicating significant potential environmental and health impacts for local residents.

The concentration distributions of all the pollutants are shown in Figure 2b. Overall, organic matter and water-soluble inorganic ions have the largest contribution to the PM_2.5_ mass. Among them, NO_3_^−^ represents the largest part of all the inorganic elements and ions detected, accounting for 62.93%. The concentration of NO_3_^−^ ranged from 0.48 to 74.32 μg/m^3^ in these three winter months, with an average value of 15.21μg/m^3^. The next is SO_4_^2−^, which varies from 0.86 to 33.35 μg/m^3^, with an average of 7.90 μg/m^3^, accounting for 32.69%. This is consistent with previous researches, which identified nitrates and sulfates as the primary inorganic components in Shanghai [40,41,42]. Simultaneously, it is also consistent with the overall trend that sulfate exhibited a decreasing trend over the past two decades, while nitrate displayed a clear increase [43]. If we focused on mainly the element, the result is displayed in Figure 2c,d. The contribution of these elements to local PM_2.5_ is in the order: Fe > Si > Ca > Mn > Pb > Ba > Cu > As > Cr > Cd > Ni > Hg, which is not exactly consistent with the results observed at other suburban sites [27,28]. The most abundant elements are Fe, Si, and Ca, together accounting for 91.01% of all the elements, indicating the regional characteristic of air pollution in the suburban area of the YRD region.

### 3.2. Health-Risk Assessment

Four heavy metals (Cr, As, Ni, and Cd) were selected to evaluate HQ and ILCR to local residents. Based on previous studies, we have conducted a unified characterization of the health risks of different genders and age groups.

According to the non-carcinogenic hazard quotient, if HQ < 1, the non-carcinogenic risk to the human body is small or can be ignored. If HQ > 1, adverse health effects might exist in residential [44]. As shown in Table S2, the non-carcinogenic risks of four heavy metals were lower than the standard, and the total value was also within the acceptable range. However, among them, Cr is the major contributor, accounting for 87.1% of the total non-carcinogenic hazard (Table S2). Ni has the negligible non-carcinogenic risk compared to other elements, accounting for only 0.1%.

The total ILCR value of four main heavy-metal elements is 9.35 × 10^−5^, which is higher than the safe risk threshold suggested by U.S. Environmental Protection Agency (10^−6^) but within the recommended risk threshold (between 10^−6^–10^−4^), demonstrating that the residents are, to a certain extent, exposed to the health risk through respiratory pathways. It is worth noting that, except Ni, concentrations of Cr, As, and Cd were all found to exceed the safe threshold for carcinogenic risks. Among the potentially carcinogenic elements under consideration, Cr posed the greatest cancer risk, accounting for 62.4% of the total cancer risks, and As was the second largest contributor (29.2%) (Table S2). As the temporal variations of the health-risk index shown in Figure S3, the ILCR value of Cr exceeded the maximum threshold in many periods, indicating great adverse health effects on the exposed residents.

### 3.3. Source Apportionment

**Figure 3:**
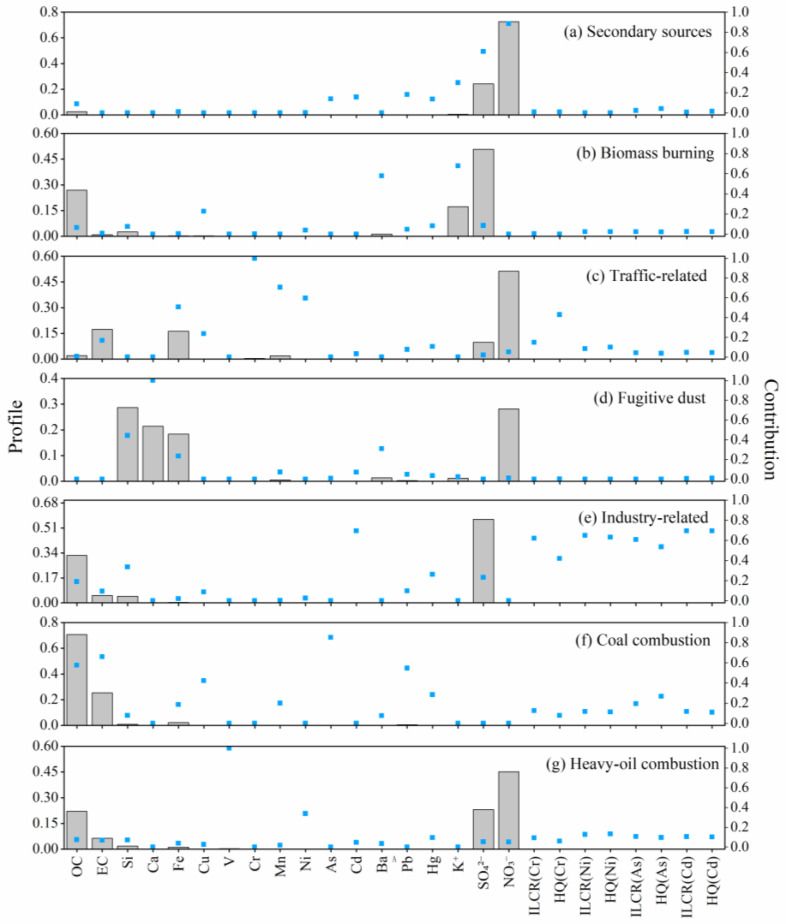
Seven factors identified by source apportionment. Factor profiles identified for all compounds and health-risk parameters (ILCR and HQ) for Cr, Ni, As, and Cd are displayed in the bar chart. The relative contribution of individual species to each factor is depicted by the blue dots.

As a suburban site, there is a combined effect of local pollution sources and surrounding pollutant transport. Seven potential sources of pollutants were identified using ME-2 and shown in Figure 3, including secondary sources, biomass burning, traffic-related sources, fugitive dust, industry-related sources, coal combustion, and heavy-oil combustion.

Factor 1 presents a high loading of NO_3_^−^ and SO_4_^2−^, which are considered to be secondary nitrate and sulfate and associated with secondary sources [45,46]. Factor 2 shows high loading of K^+^ and Ba. It is generally considered that K^+^ is mainly from biomass burning [47,48,49]. The high percentage of Ba in this factor might indicate that the main crop burning near this area is wheat straw [50], which is consistent with the dominant crop type in the surrounding areas. Jiangsu and Anhui provinces, located nearby, are the major wheat-producing regions in the middle and lower reaches of the Yangtze River in China. After the harvest season, wheat straw is usually burned for disposal during late October to November. The high concentrations of Ba detected might have originated from these burning events which occurred during the observation period and their subsequent cumulative effects. Factor 3 is composed of high loadings of Cr, and moderate Mn, Ni, and Fe, which may point to the complex traffic-related sources. Among them, Cr is associated with the emission of lubricating oil, tailpipe emissions, and road abrasion [51]. Mn, Ni, and Fe could be partially from vehicular exhausts and the combustion of lubricating oil and fuel additives [52,53]. Higher EC loading than OC found in this factor also indicates the incomplete fuel combustion associated with vehicle emissions [54]. Factor 4 presents three characteristic peaks of Ca, Si, and Fe, with a moderate amount of Ba. These are generally considered to be crustal sources and derived from fugitive dust [55]. Factor 5 was characterized by high loadings of Cd and moderate Si and Hg. Cd usually originates from high-temperature processes in industry-related activities [56], such as industrial-process emissions or industrial coal combustion [57,58,59]. Different from the Si in Factor 4, 33.5% of Si here could be from large brick factories or concrete companies in the surrounding towns. Through the characterization of different types of stack gases from stationary sources, Zeng et al. indicated that high content of Si in air can also appear near glass factories, chemical factories, and cement factories [60]. Factor 6 is characterized by a high loading of As, moderate Pd, and high values of EC and OC. The elements are usually enriched in coal [58]. Together with a high signal of organic-matter content, Factor 6 can reliably indicate coal combustion sources. Factor 7 is distinguished by a distinct high loading of V and a moderate Ni, which is considered a tracer for heavy-oil combustion, which includes the use of non-road machinery and shipping transportation [61,62].

**Figure 4:**
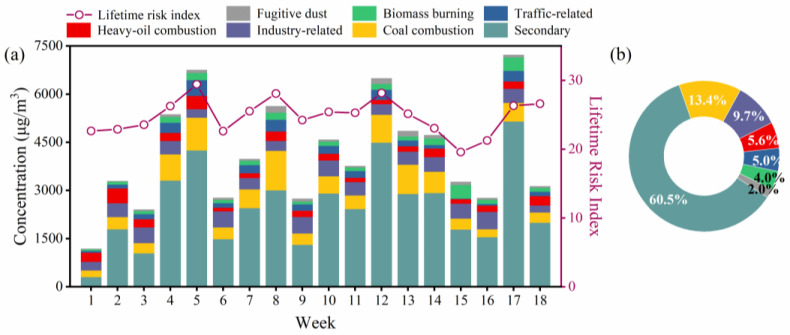
(**a**) Temporal variations of different sources. (**b**) The average contribution from the seven sources. The red line in (**a**) indicates the lifetime health-risk index, which is calculated by inhaled non-cancer hazard index (HQ) and increased lifetime cancer risk (ILCR).

The temporal and average contributions of seven identified sources are displayed in Figure 4. It can be observed that the secondary sources are the major contributors to the local atmospheric composition in every weekly statistics bar, and accounting for 60.5% of the total pollution sources. This indicated a significant influence of long-distance transport of pollutants from surrounding towns and urban areas. Important contributions from coal combustion and industry-related sources were also found, accounting for 13.4% and 9.7%, respectively. Fugitive dust was identified as the least significant contributor (2.0%). The percentage of different sources in the sampling period can be found in Figure S4.

The use of the lifetime-risk index in Figure 4 provides an intuitive visualization of the health hazards associated with heavy-metals exposure. The index is calculated as the sum of the equivalent conversed HQ and ILCR values of four heavy metals. It can be observed that the lifetime-risk index generally displays a similar trend to the concentration of pollutants, although some discrepancies existed. This might suggest that certain heavy-metal elements and sources may be primarily responsible for the adverse effects on human health.

### 3.4. Contribution of Different Sources to Health Risks

**Figure 5:**
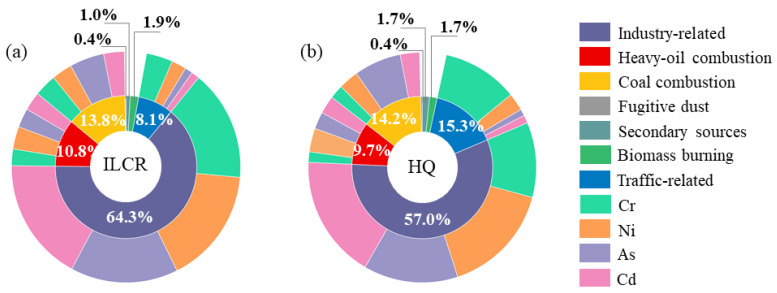
The contribution of different sources to health risks and contribution of four heavy-metal elements in each source.

While using the ME-2 model for source apportionment, ILCR and HQ were simultaneously employed to allocate the health effects to different sources and quantify their respective contributions. It can be seen in Figure 5 that among all the sources, the total ILCR and HQ of four heavy metals emitted by industry-related sources are significantly higher than those from other sources. The three major contributors to carcinogenic risks were industry-related sources (64.3%), coal combustion (13.8%), and heavy-oil combustion (10.8%). As for the non-carcinogenic risks, industry-related sources remained the major contributor of non-carcinogenic risk among all the potentially sources (57.0%), followed by traffic-related (15.3%) and coal combustion (14.2%). Heavy metals in fugitive dust have the least harm to human health compared with other sources. The detailed contribution of the seven sources can be found in Table S3.

It should be noted that, despite the relatively low contribution to the overall source apportionment (9.7%), industry-related sources have the potential to pose significant health risks to local residents. Specifically, the heavy metals emitted from surrounding industrial activities can contribute significantly to the metal-induced cancer risks faced by the local population. This is, to some extent, different from previous studies conducted in urban areas [27,63], which indicated traffic emission and coal combustion posed the predominant metal-induced health risk.

In fact, the contributions of different sources to both carcinogenic and non-carcinogenic risks associated with heavy metals exhibit regional characteristics, which can reflect the different industrial and energy structures [63]. In our study, the higher health risks associated with industry-related emissions might be due to the gradual expansion of industrialization into suburban areas caused by the relocation of factories from urban to suburban regions. Additionally, as revealed in our study, the toxicity of secondary sources may not necessarily be higher than that of industry-related emissions, although it is the largest contribution to local pollutants. Therefore, future research on the primary toxic components and their sources is necessary to better understand the specific sources of health risks and develop more effective pollution-control strategies.

## 4. Conclusions

This study evaluated the pollution sources and associated health effects of PM_2.5_ detected during a three-month winter period in the suburban area of Shanghai. Secondary sources are the major contributor to local atmospheric components, accounting for more than 60% of the total pollutants, indicating the significant influence of long-distance transport from surrounding towns and urban areas. Health-risk assessment of Cr, Ni, As, and Cd revealed the local residents were exposed to respiratory carcinogenic risks induced by heavy metal. Cr posed the predominant impact, accounting for 62.4% for ILCR and 87.1% for HQ. By combining the health-risk assessment with source apportionment using the ME-2 model, we found that industrial-related emissions, which accounted for only 9.7% of the local aerosol components, were the predominant source of health risks for the residents, contributing to 64% of the metal-induced cancer risks. This could be attributed to the gradual expansion of industrialization into suburban areas caused by the relocation of factories. Our results emphasize the correlation between health-risk assessment of the primary toxic components and pollution concentration and source contribution may require further investigation for more accurate results. The findings of our study could provide scientific guidance to develop more precise pollution-control strategies and policy improvements that better protect public health.

## 5. Uncertainty of Health Risk Assessment

While efforts were expended to ensure the accuracy and reliability of the data, we acknowledge some limitations in this study. Firstly, exposure-risk assessment involves several parameters, including *IR*, *EF*, *ED*, and *BW*. However, this study calculates the average values for all age groups and genders to represent the overall health risk to human body, which may result in some discrepancies with previous studies. Additionally, gaps and omissions of data due to various objective factors and potential confounding factors that were not accounted for in the analysis may also affect the overall value calculations. Furthermore, we only focused on the risk assessment of four major heavy-metal elements. Therefore, the characterization of health risks in this study may be incomplete.

Future research should aim to expand the scope of the analysis to include a more comprehensive range of pollutants, in order to provide a more accurate and comprehensive assessment of the health risks associated with air pollution. The method of incorporating health-risk indexes into source apportionment also requires further refinement.

## Data Availability

The data from this study have been deposited in the Zenodo data archive with the DOI https://doi.org/10.5281/zenodo.6473085 and are publicly available for research purposes [29].

## Supplementary Materials

The following supporting information can be downloaded at: https://www.mdpi.com/article/10.3390/toxics11070552/s1, Figure S1: The variation of Q/Q_exp_ with the increase number of factors; Figure S2: Scatterplot between the modeled output and the input values; Figure S3: Temporal variations of HQ and ILCR; Figure S4: Contribution of different sources; Table S1: Data statistics of the 26 species included in ME-2 analysis (ng/m^3^); Table S2: ILCR and HQ value of four heavy metals in PM_2.5_ in this study; Table S3: Contribution of seven identified sources to ILCR and HQ and the contributions of the four elements to each source.
