# Supplementary material for: Comprehensive Assessment of Pollution Sources and Health Impacts in Suburban Area of Shanghai
Source: Toxics. 2023 Jun 23;11(7):552. doi: 10.3390/toxics11070552 (PMC10383545; doi:10.3390/toxics11070552)
Supplement: Supplementary file 1 [file toxics-11-00552-s001.zip › toxics-2426165-supplementary.pdf]

Supplementary Materials of

Comprehensive Assessment of Pollution Sources and Health Impacts in Suburban Area of Shanghai

Wan Wei [1], Meng Wang [1,*], Qi Yuan [1], Zhuozhi Zhang [1], Xinwei Li [1], Shuwen Han [1], Yusen Duan [2], Qingyan Fu [2] and Shun-cheng Lee [1,*]

[1] Department of Civil and Environmental Engineering, The Hong Kong Polytechnic University, Kowloon, Hong Kong SAR, China; wanwei@polyu.edu.hk (W.W.); yq-qi.yuan@connect.polyu.hk (Q.Y.); zhuo-zhi.zhang@connect.polyu.hk (Z.Z.); xin-wei.li@connect.polyu.hk (X.L.); shuwen.han@connect.polyu.hk (S.H.)

[2] Shanghai Environmental Monitoring Center, Shanghai 200030, China; duanys@sheemc.cn (Y.D.); qingyanf@sheemc.cn (Q.F.)

* Correspondence: meng-cee.wang@polyu.edu.hk (M.W.); shun-cheng.lee@polyu.edu.hk (S.C.L.)

Table S1. Data statistics of the 26 species included in ME-2 analysis (ng/m^3).

Species	Arithmetic mean	Median	Minimum	Maximum
OC	5157.16	4003.55	799.26	25420.03
EC	1676.05	1185.16	76.79	9073.63
Si	424.68	337.34	79.06	2694.00
Ca	130.90	75.98	0.04	1317.00
Fe	476.43	361.87	14.12	2953.00
Cu	16.33	10.82	1.65	415.26
V	2.18	1.09	0.12	18.33
Cr	6.89	3.90	0.02	147.15
Mn	42.73	31.62	0.09	292.29
Ni	4.69	3.61	0.25	38.02
As	9.02	6.55	0.10	119.54
Cd	5.65	5.26	1.01	22.24
Ba	28.03	20.48	0.33	279.67
Pb	33.86	25.45	1.05	856.70
Hg	2.67	2.23	0.04	71.01
K^+	275.90	207.00	20.00	2590.00
SO_4^{2-}	7804.04	6363.50	891.00	33353.00
NO_3^-	15043.43	10464.00	480.00	74318.00
ILCR(Cr)	2.21	2.24	1.24	3.15
HQ(Cr)	7.08	6.44	1.94	38.80
ILCR(Ni)	1.61	1.61	1.36	1.93
HQ(Ni)	2.25	2.25	1.79	2.92
ILCR(As)	2.06	2.13	1.53	2.91
HQ(As)	4.20	4.25	2.39	9.18
ILCR(Cd)	1.93	1.93	1.70	2.20
HQ(Cd)	3.38	3.37	2.72	4.27

Table S2. ILCR and HQ value of four heavy metals in $PM_{2.5}$ in this study.

Elements	HQ	Percentage	ILCR	Percentage
Cr	4.85×10^{-2}	87.11%	5.83×10^{-5}	62.35%
Ni	4.57×10^{-5}	0.08%	7.91×10^{-7}	0.85%
As	6.00×10^{-3}	10.77%	2.73×10^{-5}	29.16%
Cd	1.14×10^{-3}	2.04%	7.16×10^{-6}	7.65%
Total	5.57×10^{-2}	100%	9.35×10^{-5}	100%

Table S3. Contribution of seven identified sources to ILCR and HQ and the contributions of the four elements to each source.

	ILCR			HQ		
Secondary sources	1.0%	Cr	21.84%	1.7%	Cr	14.02%
		Ni	0.00%		Ni	0.00%
		As	59.98%		As	63.00%
		Cd	18.18%		Cd	22.98%
Biomass burning	1.9%	Cr	6.58%	1.7%	Cr	0.00%
		Ni	30.92%		Ni	34.08%
		As	30.94%		As	32.25%
		Cd	31.56%		Cd	33.67%
Traffic-related	8.1%	Cr	46.36%	15.3%	Cr	70.23%
		Ni	25.93%		Ni	16.28%
		As	13.46%		As	6.12%
		Cd	14.25%		Cd	7.36%
Industry-related	64.3%	Cr	24.10%	57.0%	Cr	18.44%
		Ni	25.27%		Ni	27.66%
		As	23.63%		As	23.47%
		Cd	27.00%		Cd	30.43%
Heavy oil combustion	10.8%	Cr	21.52%	9.7%	Cr	15.15%
		Ni	29.31%		Ni	34.21%
		As	24.82%		As	24.58%
		Cd	24.35%		Cd	26.06%
Coal combustion	13.8%	Cr	22.64%	14.2%	Cr	13.75%
		Ni	21.03%		Ni	19.85%
		As	35.16%		As	47.11%
		Cd	21.16%		Cd	19.28%
Fugitive dust	0.2%	Cr	0.00%	0.4%	Cr	24.62%
		Ni	9.63%		Ni	0.66%
		As	13.04%		As	0.00%
		Cd	77.32%		Cd	74.73%

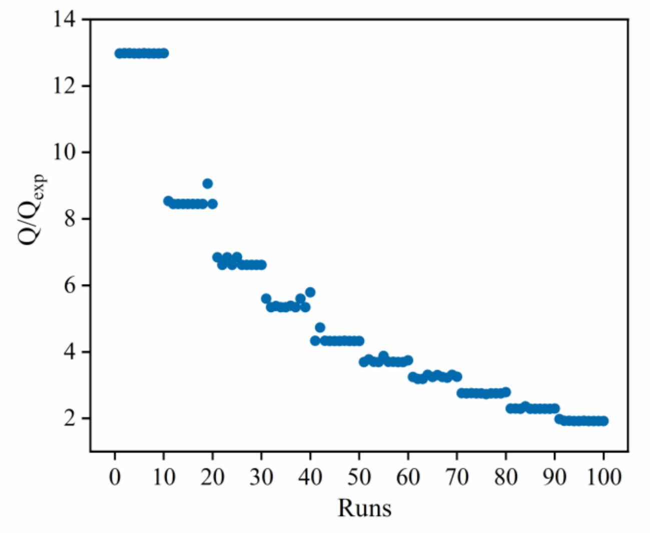

Figure S1. The variation of Q/Q_{exp} with the increase number of factors.

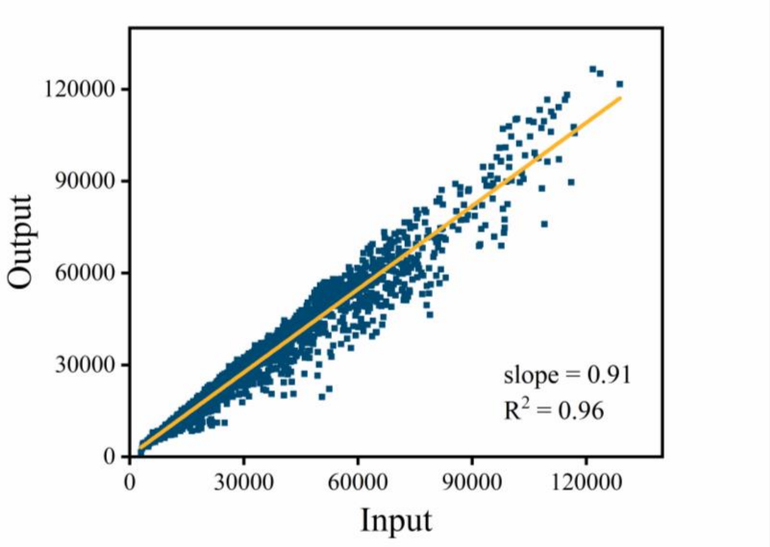

Figure S2. Scatterplot between the modeled output and the input values.

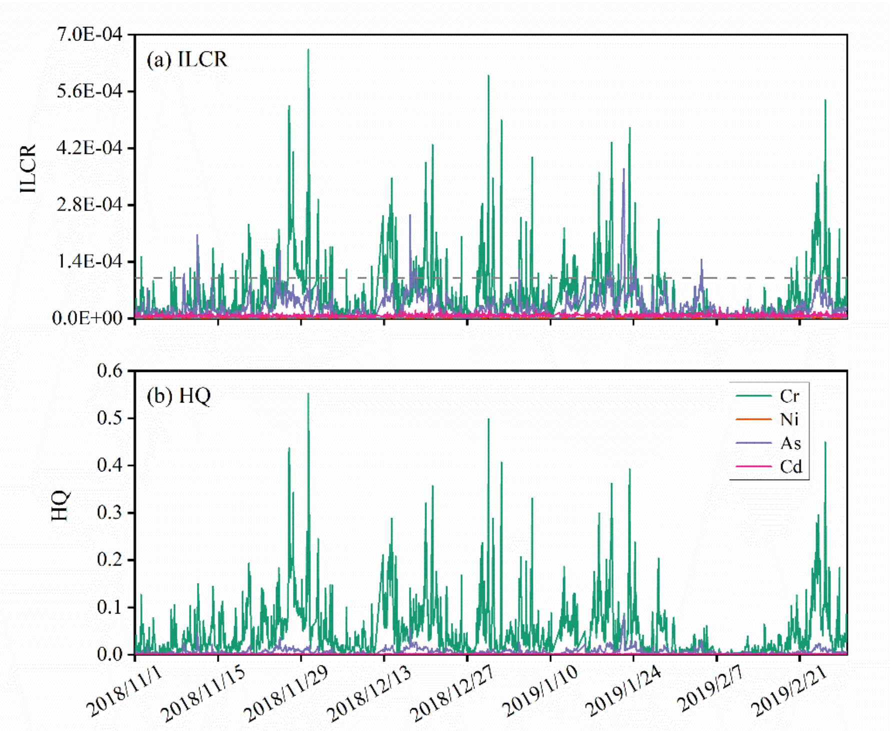

Figure S3. Temporal variations of HQ and ILCR. The two dotted lines in (a) represent the thresholds of 10^{-4} and 10^{-6}.

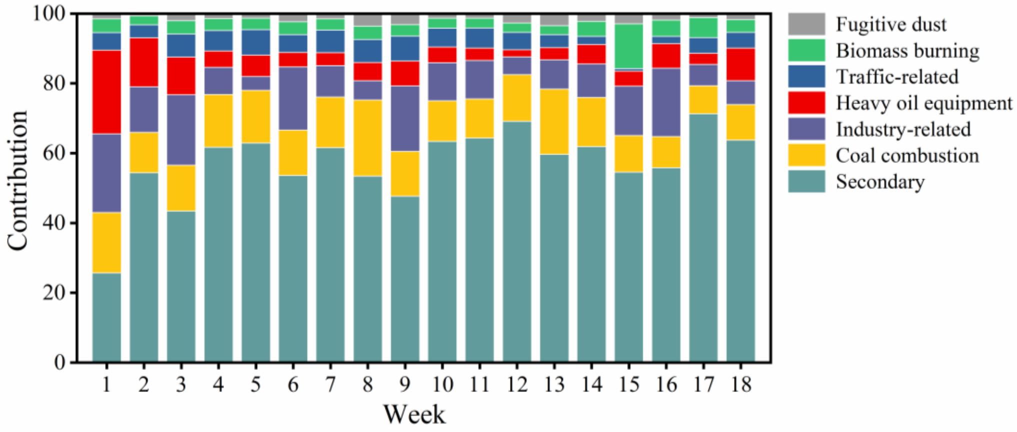

Figure S4. Contribution of different sources.
